# Re-Emergence of Dengue Serotype 3 in the Context of a Large Religious Gathering Event in Touba, Senegal

**DOI:** 10.3390/ijerph192416912

**Published:** 2022-12-16

**Authors:** Idrissa Dieng, Cheikh Fall, Mamadou Aliou Barry, Aboubacry Gaye, Ndongo Dia, Marie Henriette Dior Ndione, Amary Fall, Mamadou Diop, Fatoumata Diene Sarr, Oumar Ndiaye, Mamadou Dieng, Boly Diop, Cheikh Tidiane Diagne, Mamadou Ndiaye, Gamou Fall, Mbacké Sylla, Ousmane Faye, Cheikh Loucoubar, Oumar Faye, Amadou Alpha Sall

**Affiliations:** 1 Arboviruses and Haemorrhagic Fever Viruses Unit, Virology Department, Institut Pasteur de Dakar, Dakar 220, Senegal; 2 Epidemiology, Clinical Research and Data Science Department, Institut Pasteur de Dakar, Dakar 220, Senegal; 3 Respiratory Viruses Unit, Virology Department, Institut Pasteur de Dakar, Dakar 220, Senegal; 4 Ministry of Health, Dakar 16504, Senegal

**Keywords:** dengue virus 3, re-emergence, genotype III, Senegal, 2018, mass gathering, mobile laboratory

## Abstract

Dengue virus (DENV) was detected in Senegal in 1979 for the first time. Since 2017, unprecedented frequent outbreaks of DENV were noticed yearly. In this context, epidemiological and molecular evolution data are paramount to decipher the virus diffusion route. In the current study, we focused on a dengue outbreak which occurred in Senegal in 2018 in the context of a large religious gathering with 263 confirmed DENV cases out of 832 collected samples, including 25 life-threatening cases and 2 deaths. It was characterized by a co-circulation of dengue serotypes 1 and 3. Phylogenetic analysis based on the *E* gene revealed that the main detected serotype in Touba was DENV-3 and belonged to Genotype III. Bayesian phylogeographic analysis was performed and suggested one viral introduction around 2017.07 (95% HPD = 2016.61–2017.57) followed by cryptic circulation before the identification of the first case on 1 October 2018. DENV-3 strains are phylogenetically related, with strong phylogenetic links between strains retrieved from Burkina Faso and other West African countries. These phylogenetic data substantiate epidemiological data of the origin of DENV-3 and its spread between African countries and subsequent diffusion after religious mass events. The study also highlighted the usefulness of a mobile laboratory during the outbreak response, allowing rapid diagnosis and resulting in improved patient management.

## 1. Introduction

Dengue fever (DF) is a growing public health problem globally, and is endemic in more than 125 countries worldwide [1]. The infection may be asymptomatic or can result in a wide range of clinical symptoms ranging from a mild fever to severe forms such as dengue hemorrhagic fever [DHF] or dengue shock syndrome [DSS] [2]. Dengue virus (DENV) is a mosquito-borne virus transmitted by *Aedes* spp. and belongs to the *Flavivirus* genus, in the family *Flaviviridae*. Human transmission occurs in 128 countries where the main vectors are Aedes (Ae). aegypti and Ae. albopictus mosquitoes. DENV infections usually spread through the bites of infected competent mosquitoes [3], which spread in tropical and subtropical environments and are mostly found in urban and semiurban areas [4].

In contrast, the sylvatic transmission cycle takes place in sylvan environments, such as Southeast Asia and West Africa where the main vectors are *Ae. luteocephalus*, *Ae. Furcifer* and *Ae. Taylori* [5].

However, the other two vectors, *Ae. polynesiensis* and *Ae. niveus*, have been identified as secondary vectors in some regions throughout the world [4].

It was first isolated in 1943 in Japan, with 4 well-distinct serotypes (DENV1 to DENV4) [6] Immunity is serotype specific and does not guarantee cross protection to other serotypes. Subsequent infections by a different serotype may increase the risk of developing severe dengue forms [7]. Estimates reveal that dengue epidemics have increased around 30 times throughout the last 50 years. Around 10^8^ new cases have occurred annually in more than 100 endemic countries, putting more than 40% of the global population at risk (ca. 2.5 billion of people). Most of these cases are reported in Asia and Africa, which together bore over 80% of the global burden [8]. In Africa, the incidence of DF may be under-estimated, due to the similarities of the clinical symptoms of other endemic infections, such as malaria [9]. The first documented dengue outbreak in the continent occurred in Durban, South Africa in 1927 [10], and subsequently, numerous outbreaks have been reported [11,12].

By 8 October 2018, a preliminary biological investigation of suspected DENV cases from Touba, in western central Senegal, had confirmed dengue virus (DENV) circulation. This finding led to the rapid deployment of a multi-disciplinary team for outbreak management with the mobilization of a mobile biosafety laboratory (MBS-Lab), equipped to safely handle any type of infectious sample. The MBS-Lab was also equipped with a scalable molecular diagnostic platform, including a fully automated and portable PCR system for a rapid diagnosis [13]. Here, we report the results of this investigation including laboratory findings from this outbreak.

## 2. Materials and Methods

### 2.1. Ethical Consideration

The Senegalese National Ethical Committee of the Ministry of Health approved the surveillance protocol as a piece of research with less than minimal risk, and written consent forms were not required. Oral consent to participate was obtained from all patients or parents/guardians of minors included in this study, as required by the Senegalese National Ethical Committee of the Ministry of Health. Throughout the study, the database was shared with the Epidemiology Department at the Senegalese Ministry of Health and Prevention for appropriate public health action.

### 2.2. Real Time Case Notification and MBS-Lab Deployment

**Figure 1:**
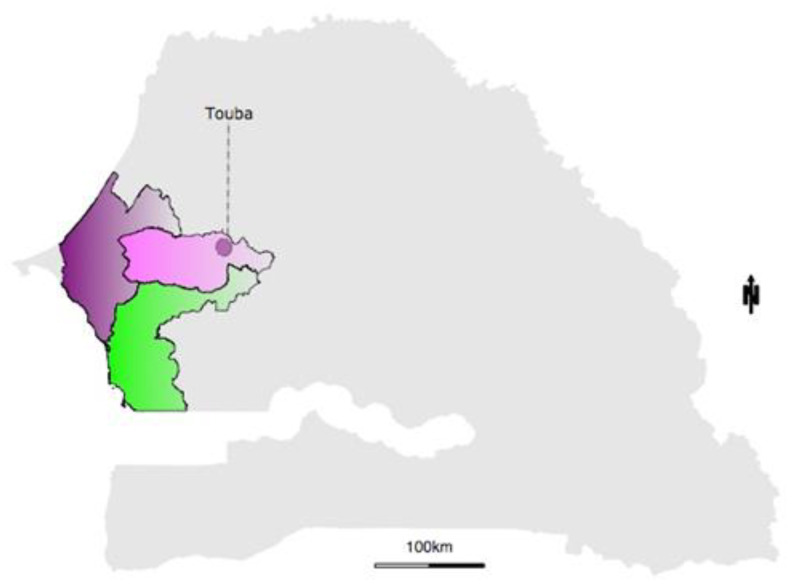
Map showing the Touba (pink) area where the DENV-3 outbreak occurred. In the same period, some sporadic DENV cases were reported in Thiès (Purple) and Fatick (Green).

There was a notification of increasing cases of fever in the city of Touba through the sentinel surveillance network in Senegal (4S network). On 8 October 2018, blood samples were collected from suspicious patients and sent to the Department of Virology of the Institut Pasteur de Dakar (IPD) for further investigation and a determination of the potential associated pathogen. At the IPD, blood samples were centrifuged at 2000 rpm for 5 min for sera/plasma collection and viral RNA was extracted using the Qiagen RNA kit according to the manufacturer’s instructions. The resulting RNA was screened by targeting the major arboviruses commonly circulating in Africa, including dengue virus (DENV), chikungunya virus (CHIKV), yellow fever virus (YFV), zika virus (ZIKV) and Rift Valley fever virus (RVFV), according to a protocol previously described [14]. From the performed molecular assays, only DENV gave a positive result. This led to the notification of DENV circulation during the timeframe of the huge religious mass gathering event, the Grand Magal de Touba (Figure 1). The Senegalese Ministry of Health (MoHS) raised concerns about the urgency of screening suspected dengue cases at the point of need in order to contain the spread and allow for early case detection and management. The MBS-Lab, which previously showed utility in epidemic response [15], was deployed in Touba on 17 October 2018 upon the request of the MoHS.

### 2.3. Patient Enrolment and Sample Collection

In keeping with standard WHO case definitions, DF can be stratified into 3 different categories according to clinical severity. These are: (i) Dengue without warning signs (DWiWS), defined as a suspicious dengue case with acute fever (>38.5 °C) and at least two of the following symptoms: headache, retro-orbital pain, nausea/vomiting, muscle and joint pains, rash, petechiae or leukopenia; (ii) Dengue with warning signs (DWWS), defined as a dengue case with 1 or more of these signs: intense abdominal pain, persistent vomiting, fluid accumulation (ascites, plural, and/or pericardial effusion), mucosal bleeding, lethargy, lipothymia, liver enlargement, or progressive increase in hematocrits; and (iii) Severe dengue (SD) defined as a dengue case with 1 or more of the following manifestations: defined shock or respiratory distress (DSS), severe bleeding or severe organ compromise. The last two categories can be fatal and require strict observation and medical intervention [8]. For each patient visiting healthcare centers across Touba and matching inclusion criteria, 5 mL of venous blood was collected into a 5 mL tube. Collected samples with a standardized form filled with epidemiological and clinical data were transported to the MBS-Lab located at Djemoul healthcare center, where confirmation tests were achieved. Any suspected cases with a positive result by qRT-PCR dengue and/or IgM signal confirmed by plaque reduction neutralization assay was considered as a confirmed case.

### 2.4. RNA Extraction

A total of 832 samples were collected from suspected DENV patients between 1 October 2018 and 16 November 2018 from healthcare centers across Touba. Viral Ribonucleic Acid (RNA) was extracted from 140 µL of sera using the Qiagen (Qiagen, Hilden, Germany) viral RNA kit according to the manufacturer’s recommendations. RNA was then eluted in 60 µL of molecular grade water and stored at −20 until further use.

### 2.5. Dengue Virus Detection by qRT-PCR

In order to confirm acute dengue infection, qRT-PCR was performed on extracted RNA. The molecular detection was performed on patients with a fever history of at least 7 days using the set of primers described by Wagner and colleagues [16]. The system targeting the 3′-UTR region of DENV allowed for detection of all serotypes. The reaction was performed inside the MBS-Lab with a Smartcycler thermocycler (Cepheid, Sunnyvale, CA, USA) using Quantitect kit (Qiagen, Hilden, Germany). Briefly, the viral RNA detection was performed using the following temperature profile: reverse transcription at 50 °C for 10 min, initial denaturation at 95 °C for 15min, followed by 45 cycles of denaturation at 95 °C for 15 s, and 60 °C for 1min. Samples with Ct values <37 were considered as positives.

### 2.6. Dengue Virus Antibody Detection by Serological Assays

Serological testing was carried out on samples from suspicious patients at the end of the acute phase by enzyme-linked immunosorbent assay (ELISA) IgM and by 90% plaque-reduction neutralization test (PRNT) for confirmation, respectively. For IgM detection, plates were coated with monoclonal capture antibody (anti-human IgM), incubated overnight at +4 °C and then washed at least twice with wash buffer (1× phosphate-buffered saline (PBS) supplemented with 0.05% Tween-20) and tap dried. After blocking with freshly prepared blocking buffer (1× PBS with 5% of nonfat dry milk), plates were incubated at room temperature for 2 h, washed twice and tap dried. Sera were heat-inactivated (56 °C for 30min) and diluted (1:100) in a 1× PBS and added to the test wells, in addition to control dengue antigens, and incubated at room temperature for 2 h. Detection was done using added substrate solution (TMBS) and incubated in a humidified box protected from light, which was further stopped after sufficient coloration by adding sulfuric acid. Absorbance was read with a plate reader (spectrophotometer) at the wavelength of 492 nm. The results were interpreted by comparing test OD with positive and negative controls with a cut-off of 0.2 [17].

On IgM positives samples, the 90% plaque-reduction neutralization test (PRNT90) was performed to determine the maximum serum dilution (1:8 to 1:1024) needed to reduce arbovirus plaque formation by 90% among Vero cells. The cut-off value for PRNT positivity was defined as 90% (PRNT90). Thus, the DENV-2 strain New Guinean C was used. Correspondingly, previously heat inactivated sera were serial diluted with Gibco Dulbecco’s Modified Eagle Medium (DMEM) containing 2% fetal calf serum and 1% of Penicillin/Streptomycin. Virus suspension was mixed with each serum dilution and incubated at 37 °C for 60 min before their transfer to Vero cells and additional incubation at 37 °C for 60 min. Next, 0.3% agarose solution was added, and the plates were incubated at 37 °C for 3–5 days. Reactions were then revealed using a 2% naphthol blue-black solution. Titers ≥10 were considered as positive.

### 2.7. Data Management and Real Time Case Reporting

In order to allow for the early reporting of confirmed dengue cases and the real time monitoring of epidemic evolution, as well as the identification of hotspots, a web-based platform was developed, in collaboration with the epidemiological team. This application, called ‘’Teranga” (https://teranga.pasteur.sn// (daily during investigation period)) written in php (Backend); html, javascript (Frontend) can be accessed anywhere using a laptop, tablet or smartphone connected to internet. Basically, the platform provides an online form where any type of data, including epidemiological as well as clinical data, can be registered and linked to a unique identifier. The results can be exported as an Excel file with the possibility to represent them as graphs (bar plot, epidemic curves, map of confirmed cases) that are updated in real time. This offers daily access to the healthcare giver to the DENV results of the enrolled patients in their respective health districts.

### 2.8. Complete E Gene Sequencing

To get insight about the origin of the DENV-3 strain mainly responsible for the epidemic, the *E* gene of a random selection of 17 positive samples was sanger sequenced. cDNA synthesis was performed using AMV kit (Promega, Madison, WI, USA). Briefly, 10 µL of the extracted RNA of viral RNA was mixed with 1 μL of the random hexamer primer (2 pmol) and the mixture was heated at 95 °C for 2 min. Reverse transcription was performed in a 20 μL reaction mix containing 2.5 U RNasin (Promega, Madison, USA), 1 μL of deoxynucleotide triphosphate (dNTP) (10 mM each DNTP), and 5 U of AMV reverse transcriptase (Promega, Madison, USA), by incubating it at 42 °C for 60min. PCR products were generated using the sets of primers described previously [18], which allow for the amplification of overlapping fragments of the full E gene of DENV-3. A quantity of 5 µL of cDNA was mixed with 10 μL of 10× buffer, 3 μL of each primer, 5 μL of dNTPs 10 mM, 3 μL of MgCl_2_, and 0.5 μL of GoTaq polymerase (Promega, Madison, WI, USA). The obtained amplicons were purified using a QIAquick Spin PCR Purification kit (Qiagen, Hilden, Germany) and submitted for bidirectional sequencing, then sent for bi-directional sequencing using an ABI 377 automated sequencer (Applied Biosystems, Waltham, MA, USA) using the same PCR primer set used for PCR reactions. The raw data were edited and merged using Geneious Prime to get the complete *E* gene sequences.

### 2.9. Dataset Phylogenetic and Phylogeographic Analysis

In addition to the 11 successfully completed *E* genes obtained during our study, the complete *E* gene sequences of DENV-3 available in Genbank, with complete metadata, (country of isolation and date of isolation) were downloaded from the National Center of Biotechnology Information (NCBI) in Genbank format and then converted to fasta using a custom perl script. The sequences were then filtered to exclude identical sequences (https://biopython.org/wiki/Sequence_Cleaner, accessed on 9 March 2021) resulting in a non-redundant set, namely dataset-1. This primary dataset was then used to run a maximum likelihood tree using FASTTREE. From this initial tree, a cluster of viral strains closely related to our isolates were extracted to get a dataset-2 of 40 sequences used for Bayesian inference). For each described dengue genotype among DENV-3 serotype, four to ten sequences were selected and combined with Senegalese DENV-3 sequences (dataset-3) for the purpose of genotyping. Phylogenetic analysis was conducted on dataset-3 using the maximum likelihood (ML) phylogenetic approach implemented in IQ-TREE v.1.5.5 software [19] with an automatic model selection conducted by ModelFinder (MF) according to the Bayesian information criterion (BIC). The robustness of the tree topology was tested during 1000 non-parametric bootstrap analyses. The final tree was visualized and plotted using FigTree v.1.4.3 (http://tree.bio.ed.ac.uk) (accessed on 9 March 2021). All sequence data used in this work are presented in the following format: accession number_region of sampling_date of isolation.

For phylogeographic inference, the temporal signal (i.e., molecular clock structure) was investigated on dataset-2 using Tempest v.1.5.3 [20]. The spatiotemporal spread of DENV-3 during this outbreak was reconstructed under a Bayesian framework. A time-scaled Bayesian phylogenetic analysis was performed using a Markov Chain Monte Carlo (MCMC) algorithm implemented on BEAST v1.10.4 [19]. Briefly, the BEAST analysis was run for 50 million MCMC steps using a GTR+G substitution model, an uncorrelated relaxed clock with log-normal distribution and a Skyline tree prior. Trees were sampled every 5000 generations; the convergence was assessed using Tracer v1.7.1 [21] and TreeAnnotator v1.10.4 [22] was then used to generate maximum clade credibility trees with a burn-in value setting of 10%. The tree was visualized and annotated using Figtree v1.4.4 (http://tree.bio.ed.ac.uk/software/figtree/) (accessed on 9 March 2021).

## 3. Results

### 3.1. Epidemiology, Demographic and Clinical Characteristics

**Figure 2:**
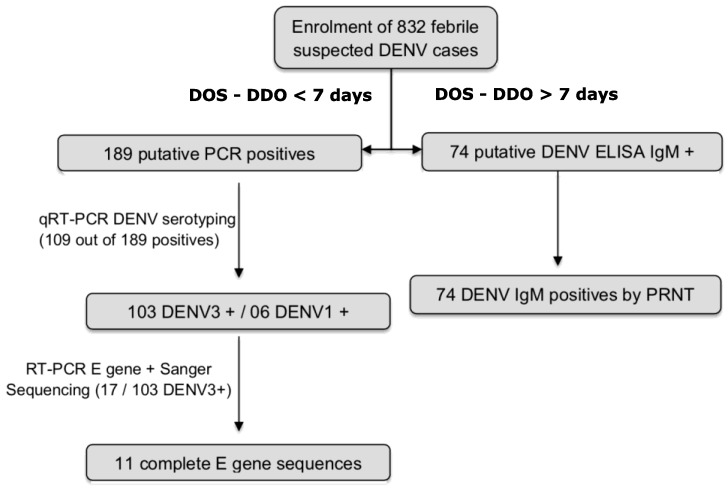
Algorithm of patient enrolment and obtained results. DDO means Date of Disease Onset and DOS means Date of Sampling.

A total of 832 sera samples were collected from 22 healthcare districts around Touba for dengue diagnosis (Figure 2). 

**Figure 3:**
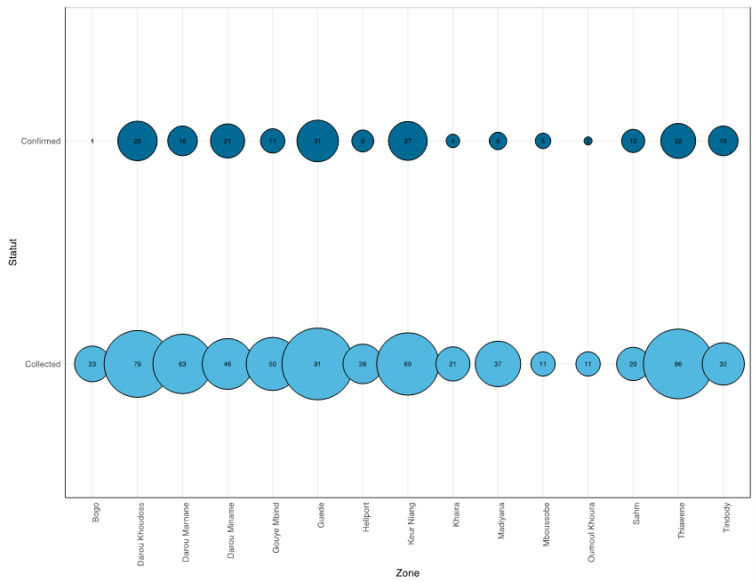
Repartition of suspected and confirmed cases according to healthcare district. We represent only districts with more than 10 suspected DENV cases. Dark blue circles represent the confirmed cases (qRT-PCR and/or IgM detection followed by PRNT) and light blue circles represent the suspected cases. The size of the circles is proportional to the number of cases.

Most of the suspected cases were collected in Guede district, with 122 suspected cases, followed by Thiawene, with 108 cases. In contrast, Oumoul Khoura and Moussobe recorded the lowest number of suspected cases with, respectively, 16 and 13 cases. The overall infection rate was 31.61%; among confirmed cases the highest number were recorded in Guede (n = 31), followed by Darou Khoudoss (n = 28), Keur Niang (n = 27) and Thiawene (n = 22) (Figure 3). 

The sex ratio M/F for the confirmed cases was 1.02; there was no statistically significant difference between positivity and gender observed (*p* = 0.111; Pearson’s χ^2^-test). According to age, most of the confirmed cases belong to the 15–30 age group (45.8%), followed by the <15 years (36.3%), with the lowest positivity group to the virus recorded in the age group 45–80 (5.2%); the rate of positivity varied significantly according to the age group (*p* < 0.001; Pearson’s χ^2^-test) (Table 1). Among positives cases, 25 patients presented with severe life-threatening symptoms, including hemorrhagic signs (n = 15; 5.7%), meningoencephalitis (n = 8; 3%) and shock syndrome (n = 2; 0.8%) (Table 1).

**Figure 4:**
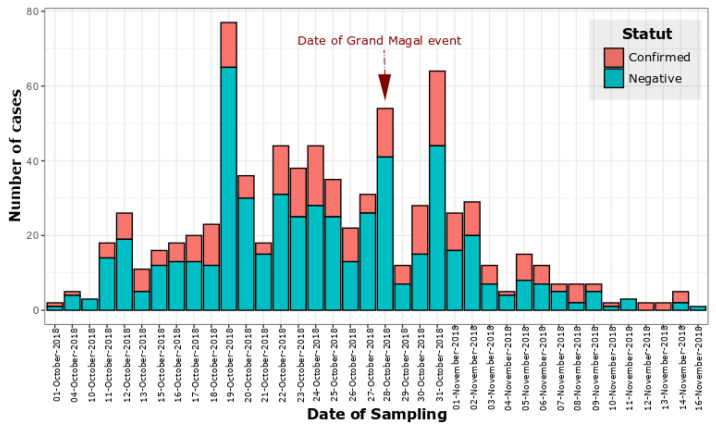
Epidemic curve describing the distribution of negatives and confirmed dengue cases according to the day of sampling. The date of the religious event (28 October 2018) is marked by the red arrow; this date corresponds to week 44 (Figure S1).

The outbreak began at epidemiological week 40 and ended at epidemiological week 46; the highest number of cases was recorded in week 44 (75 confirmed cases) (Figure 4 and Figure S1).

### 3.2. Detection of DENV RNA and Serotyping

Among 832 enrolled suspected cases during this study, 189 (22.71%) yielded positive dengue results (qRT-PCR) (Figure 4). The serotyping of 109 out of all the DENV RNA positive samples using real time PCR confirmed that the mainly circulating serotype during this epidemic was DENV-3 (103/109; 94.49%) (Figure 2).

### 3.3. Detection of DENV IgM

A total of 74 samples (8.89%, n = 832 enrolled patients) were positive for DENV IgM antibodies by ELISA. Due to the existing cross reactivity between flaviviruses, all of them were confirmed to harbor DENV specific IgM antibodies by PRNT.

### 3.4. Phylogenetic and Bayesian Analysis of Detected DENV-3 Strain

**Figure 5:**
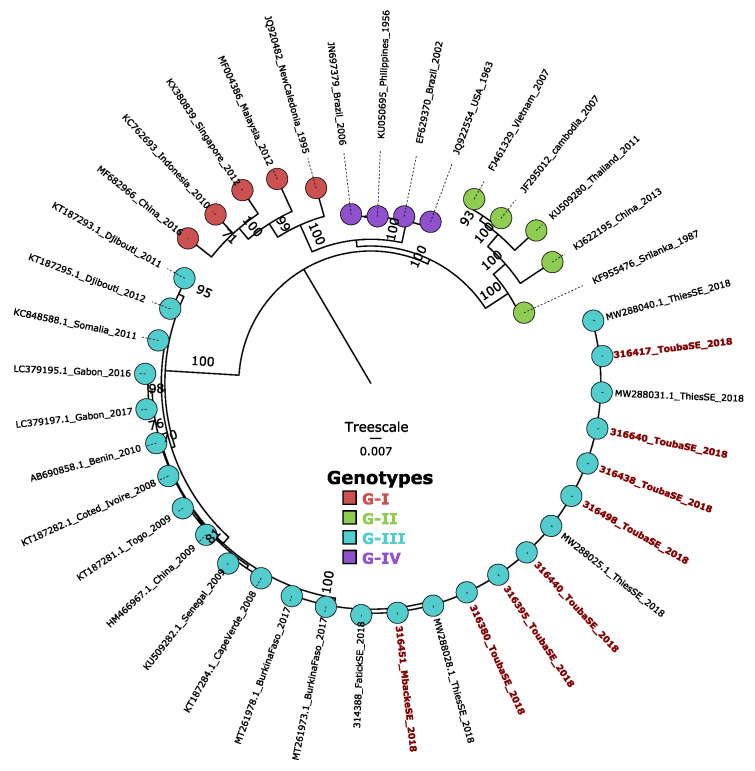
Maximum likelihood (ML) phylogenetic tree inferred by using non-identical sequences from the Touba outbreak (highlighted in red) and those from other regions of Senegal (Thiès and Fatick, sampled in 2018) in combination with strains representative of the described DENV-3 genotypes. Bootstrap support (greater than 75) is represented on each node; scale bar represents the number of observed substitutions per site. Genotypes are represented by different colors according to the legend.

Successfully recovered full-length envelope DENV-3 sequences during this work (n = 11) were combined with a representative dataset of global DENV-3 sequences and subjected to phylogenetic analysis, which revealed that the strain detected during this investigation belonged to DENV-3 genotype III; these isolates were closely related to strains collected in Thiès in 2018, Fatick in 2018 and Burkina Faso in 2017 (ML tree) (Figure 5).

**Figure 6:**
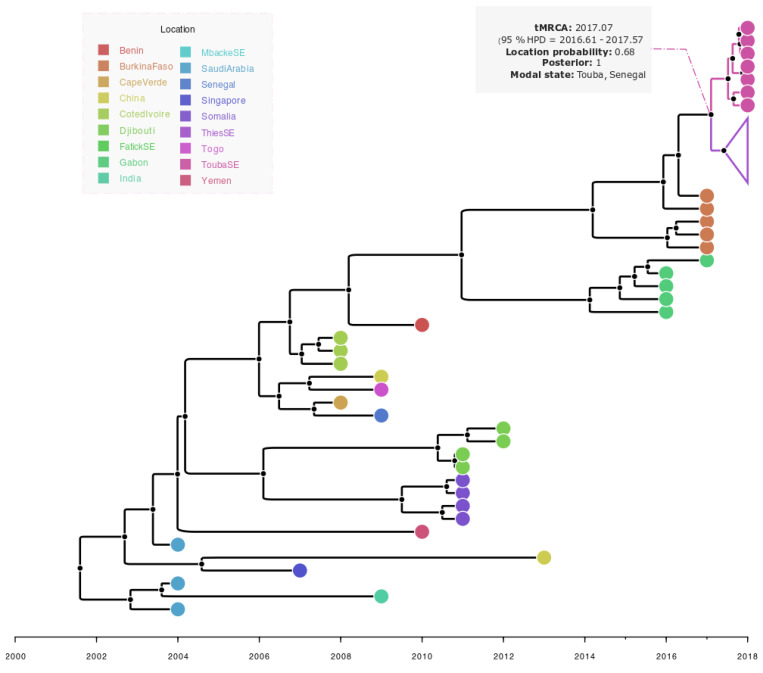
Bayesian discrete phylogeography of dengue—3 strains isolated in Touba in 2018. Touba’s strains (ToubaSE) grouped in a monophyletic cluster with sequences from Thiès (ThiesSE) (Collapsed in purple).

The performed phylogeographic analysis of sequenced viruses revealed that isolates form a monophyletic cluster which originated in Burkina Faso and were introduced in Touba, Senegal (location probability = 0.68; posterior = 1). It also suggests a single introduction possibly took place around 2017.07 (95% HPD = 2016.61–2017.57) and then spread to the Thiès region (Figure 6).

## 4. Discussion

Dengue occurrence in Senegal has long been linked to the circulation of sylvatic strains mainly in the south-eastern part of the country [20]. In 2009, a major shift occurred with the notification of the first urban dengue outbreak associated with 196 confirmed cases and affecting Dakar, Thiès and Louga; this outbreak was mainly caused by a DENV-3 serotype never detected before in West Africa [12]. Unprecedented recurrent outbreaks and sporadic cases marked by the co-circulation of three serotypes DENV1-3 have been reported in Senegal since 2017 [22]; the virus has recently emerged as a significant public health problem in Senegal. In October 2018, a dengue outbreak occurred in Senegal, more precisely at the religious city of Touba during the 124th edition of the “Grand Magal of Touba”. This event is one of the largest religious mass gatherings in Senegal with approximately 4–5 million pilgrims from Senegal and worldwide [21]. To allow the rapid containment of this outbreak in the context of a mass gathering of people, and the rapid detection of suspected cases which impacts patient management, a mobile biosafety laboratory was deployed in the holy city of Touba using the same workflow described by Dieng and colleagues during a dengue outbreak investigation in Louga in 2017 [15]. Results from the epidemiological investigation revealed that the epidemic spanned a period of 7 weeks (from week 40 to week 46) with the highest number of confirmed cases recorded in week 44; the dengue positivity rate was 31.61% with 263 confirmed dengue cases (qRT-PCR and/or IgM detection) among 832 enrolled patients. The observed positivity rate is higher than those reported from a previous DENV1 outbreak in Louga in 2017. In addition to the highest density of people in Touba during the Magal event, which is known to be a risk factor of an increased number of cases during an infectious disease outbreak [23], the observed difference can be linked to the fact that, in contrast to this study, during the Louga outbreak only molecular testing of suspected DENV cases was used, leading to a probable underestimation of real number of cases by missing IgM positive cases. A previous study revealed that PCR and capture of IgM/IgG ELISA in combination had the sensitivity to detect above 90% of cases through the course of the illness [24]. According to the age group, the incidence is significantly higher among individuals of ages 15 to 30 (*p* < 0.001; Pearson’s χ^2^-test). It corresponded to the cohort of patients attending school or in their early career; the higher dengue positivity in this age group may be due to their active involvement in socio-economic activities [25] which may increase the chance of being exposed to mosquitoes vectors.

Performed molecular serotyping using qRT-PCR showed that during this outbreak the main circulating serotype was DENV-3 (n = 103); only six case of DENV-1 were detected among successfully serotyped DENV-positive sera. Phylogenetic analysis based on the full length *E* gene shows that the strains belong to the African cluster of DENV-3 genotype III (Figure 5) which they share with strains isolated in many African countries such as Gabon, Burkina Faso, Togo, Benin, and Cote d’Ivoire, while the DENV-1 isolates detected in Touba were previously studied and belong to the genotype V [26]. Interestingly, during this study, we noticed 25 severe life-threatening cases associated with two deaths, supporting previous findings reporting an increased number of severe infections associated with the emergence of DENV3 genotype III (DENV3/III) in the Americas and Asia [27]. The extent of the outbreak was probably underestimated, as dengue infection may be asymptomatic or misdiagnosed as malaria. Furthermore, all districts did not correctly notify suspected cases and only patients with serious illness went for medical consultation. The proportion of severe dengue (SD) was slightly higher (5.7%) when compared to the reported rate (3%) during the 2009 Dengue outbreak in Senegal [12]. Overall, the high rate of SD in Senegal compared to the current situation in the Americas (from 0.26% to 1.81%) is worrying. Since prior exposition to DENV was not investigated during this work, it is possible that these patients with severe manifestations were infected earlier with a different dengue serotype.

According to previous reports, DENV-3 was very common in West and Central Africa during this last decade [12,28,29]. In Africa, the first detection of this serotype took place in Mozambique in 1985 [30]; DENV3 has been known to be endemic [31,32] and actively circulating in West Africa since 2006 [28]. Studies suggest a significant association of DENV-3 with severe cases [33], increasing the need for better monitoring of the serotype’s dissemination through pan-African genomic surveillance of circulating DENV serotypes. Interestingly, results from the phylogeographic analysis revealed that the DENV3 was introduced to Touba, Senegal (location probability = 0.68; posterior = 1), from Burkina Faso (West Africa) (Figure 6), where dengue outbreak was marked by the co-circulation of DENV2 and DENV3 reported in 2016 [29]. The introduction of the virus to Touba occurred around 2017.07 (95% HPD = 2016.61–2017.57) which corresponds to January 2017; thus, estimates in combination with the time of notification of the first DENV cases in Touba (8 October 2018) support a probable cryptic DENV3 transmission in this region prior to case notification. After its introduction in Touba, the same strain probably spread to the Thiès region located in the western part of the country (Figure 1) where it caused the outbreak in co-circulation with DENV1-2 in late 2018 [34]. Indeed, in their study, Gaye and colleagues revealed that 45% of DENV3 positive patients reported their travel to Touba during the Grand Magal religious pilgrimage. This, in addition to the provided genomic data during our study, supports the idea that the virus likely spread to Thiès, where it caused the outbreak in late 2018, through a viremic traveler or infected mosquitoes, after its introduction to Touba in 2017. Interestingly, based on the complete genome by Gaye and colleagues [34], an estimated TMRCA was provided of the Thiès sequences sampled in 2018 and the most closely related African sequences from a Gabonese outbreak sampled in 2016–2017, which was around 13 years ago, highlighting the absence of most of the recent evolutionary history, probably due to the fact that they only used seven African complete genome sequences at the time of their analysis, and no DENV3 sequences from Burkina Faso 2017 and Senegal 2018 (Touba) were available. With the addition of the DENV3 sequences from Burkina Faso 2017 and newly obtained sequences from Touba (n = 07), our study, based on complete *E* gene sequences, fine tunes the resolution by providing a TMRCA of recent Senegalese DENV3 sequences from around 3.83 years ago (95% HPD 2.07–5.72 years) (Touba and Thiès 2018) with the most closely related available sequences from Burkina Faso sampled in 2017.

Galloping urbanization and population growth in Touba, together with sanitation and water challenges and the movement of populations represent many risk factors that may cause the outbreak. Therefore, dengue surveillance and preparedness should be reinforced in Senegal, particularly in Touba during the Grand Magal preparations, which coincide with the period of DENV circulation in Senegal.

## 5. Conclusions

This work adds pieces of evidence about the growing DENV burden in Senegal; it highlighted the reliability of the MBS-Lab and its usefulness during the outbreak response in remote areas. Additionally, it reveals the re-emergence of DENV-3 in Senegal nine years after the last described epidemic caused by DENV-3 in 2009; alarmingly, in a context of a mass gathering, such as “the Grand Magal religious event”, a higher likelihood of the virus spread to other regions following the event is plausible; this calls for improved dengue surveillance around the Senegalese country and the consideration of DENV as a potential etiological agent by healthcare givers in the case of febrile illness.

## Figures and Tables

**Table 1:** Epidemiologic and clinical characteristics of suspected and confirmed dengue cases, Touba, Senegal, 2018.

	Confirmed Cases (N = 263)	Negative (N = 569)	Total (N = 832)	*p* Value
**Age**				<0.001 ^1^
Median	18	21	20	
Q1, Q3	9.5, 26.0	12.0, 32.0	12.0, 30.0	
**Group age, in year**				<0.001 ^2^
Unknown	12	27	39	
<15	91 (36.3%)	165 (30.4%)	256 (32.3%)	
(15–30)	115 (45.8%)	204 (37.6%)	319 (40.2%)	
(30–45)	32 (12.7%)	114 (21.0%)	146 (18.4%)	
(45–80)	13 (5.2%)	59 (10.9%)	72 (9.1%)	
**Sex**				0.111 ^2^
F	130 (49.4%)	315 (55.4%)	445 (53.5%)	
M	133 (50.6%)	254 (44.6%)	387 (46.5%)	
**Clinical signs and Symptoms**				
Fever	213 (81.0%)	369 (64.9%)	582 (70.0%)	<0.001 ^2^
Join pain	154 (58.6%)	326 (57.3%)	480 (57.7%)	0.732 ^2^
Asthenia	149 (56.7%)	347 (61.0%)	496 (59.6%)	0.237 ^2^
Headache	243 (92.4%)	495 (87.0%)	738 (88.7%)	0.022 ^2^
Retrorbital pain	60 (22.8%)	123 (21.6%)	183 (22.0%)	0.698 ^2^
Bleeding	15 (5.7%)	19 (3.3%)	34 (4.1%)	0.109 ^2^
Muscle pain	133 (50.6%)	311 (54.7%)	444 (53.4%)	0.272 ^2^
Abdominal pain	73 (27.8%)	168 (29.5%)	241 (29.0%)	0.601 ^2^
Meningo-encephalitis	8 (3.0%)	11 (1.9%)	19 (2.3%)	0.320 ^2^
Shock syndrome	2 (0.8%)	0 (0.0%)	2 (0.2%)	0.037 ^2^
Rash	10 (3.8%)	16 (2.8%)	26 (3.1%)	0.445^2^
**Profession**				0.256 ^2^
Bureaucrat	21 (8.0%)	52 (9.1%)	73 (8.8%)	
Trade-market	17 (6.5%)	54 (9.5%)	71 (8.5%)	
Farmer	6 (2.3%)	21 (3.7%)	27 (3.2%)	
Student	68 (25.9%)	131 (23.0%)	199 (23.9%)	
Homemaker	55 (20.9%)	142 (25.0%)	197 (23.7%)	
Unspecific activities	73 (27.8%)	128 (22.5%)	201 (24.2%)	
Workers	23 (8.7%)	41 (7.2%)	64 (7.7%)	

^1^: stands for the Kruskal-Wallis test, ^2^: stands for χ^2^-test or Fisher’s exact test.

## Supplementary Materials

The following supporting information can be downloaded at: https://www.mdpi.com/article/10.3390/ijerph192416912/s1, Figure S1: Weekly Epidemic curve of suspected and confirmed dengue cases during the outbreak investigation in Touba, 2018.
