# Supplementary material for: Re-Emergence of Dengue Serotype 3 in the Context of a Large Religious Gathering Event in Touba, Senegal
Source: Int J Environ Res Public Health. 2022 Dec 16;19(24):16912. doi: 10.3390/ijerph192416912 (PMC9779395; doi:10.3390/ijerph192416912)
Supplement: Supplementary file 1 [file ijerph-19-16912-s001.zip › ijerph-2040187-supplementary.pdf]

Article

Re-emergence of Dengue serotype 3 in the context of a large religious gathering event in Touba City, Senegal

Idrissa DIENG [1*], Cheikh Fall[1], Mamadou Aliou Barry[2], Aboubacry Gaye[2], Ndongo Dia[3], Marie Henriette Dior Ndione[1], Amary Fall[3], Mamadou Diop[2], Fatoumata Diene Sarr[3], Oumar Ndiaye[1], Mamadou Dieng[4], Boly Diop[4], Cheikh Tidiane Diagne[1], Mamadou Ndiaye[4], Gamou Fall[1], Mbacké Sylla[4], Ousmane Faye[1], Cheikh Loucoubar[2], Oumar Faye[1] and Amadou Alpha Sall [1]

[1] Arboviruses and Haemorrhagic Fever Viruses Unit, Virology Department, Institut Pasteur de Dakar, Dakar, Senegal
[2] Epidemiology, Clinical Research and Data Science Department, Institut Pasteur de Dakar, Dakar, Senegal
[3] Respiratory viruses Unit, , Virology Department, Institut Pasteur de Dakar, Dakar, Senegal
[4] Ministry of Health, Dakar, Senegal
* Correspondence: Idrissa.DIENG@pasteur.sn ; +221 761912447

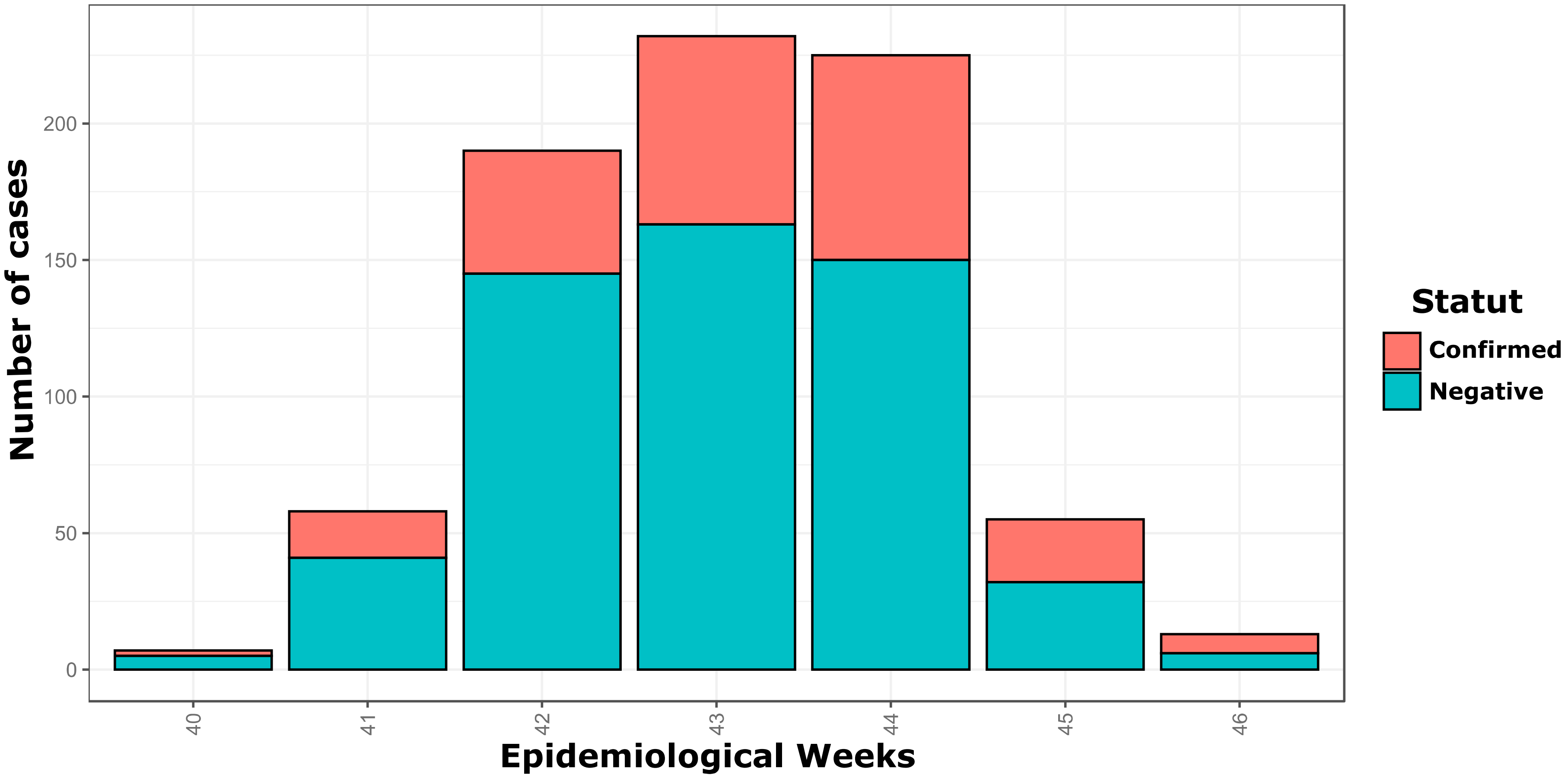

Figure S1 : Distribution of suspected and confirmed DENV human cases according to epidemiological weeks in Touba City, 2018.
